# Ethics and IVF add-ons: We need to talk about it

**DOI:** 10.5935/1518-0557.20220030

**Published:** 2022

**Authors:** Daniela Paes de Almeida Ferreira Braga, Amanda Souza Setti, Edson Borges Jr.

**Affiliations:** 1 Fertility Medical Group, Av. Brigadeiro Luis Antonio, 4545, São Paulo - SP, Brazil. Zip: 01401-002; 2 Sapientiae Institute - Centro de Estudos e Pesquisa em Reprodução Humana Assistida, Rua Vieira Maciel, 62, São Paulo - SP, Brazil. Zip: 04503-040

**Keywords:** IVF adjuncts, add-on, adjuvants, IVF, survey

## Abstract

*In vitro* fertilization (IVF) ‘add-ons’ are adjunct treatments used in addition to standard IVF protocols, in an attempt to improve success rates. However, the benefits for add-ons are often not supported by high-quality evidence. Nevertheless, many infertile patients are willing to try anything that might help them to improve their chances of having a baby. Therefore, the use of add-ons has been widespread and has led to extensive debate and discussion. The goal of this manuscript was to discuss the ethics underling the use of adjunct therapies in clinical practice before their safety has been thoroughly ascertained. IVF patients are routinely offered and charged for a wide range of adjunct treatments that they are told may improve their chance of a live birth, despite there being no clinical evidence supporting such efficacy. Add-on treatments are well accepted by most infertile patients, especially those who have already started their IVF treatments. A particular concern is that many clinics around the world are advertising and offering clinical adjuncts to infertile couples undergoing IVF, however, information on add-ons is often inaccurate. Data concerning the lack of scientific evidence supporting add-on efficacy and whether an add-on may cause unanticipated harm or worsen treatment outcomes is not available on most websites. IVF patients are a vulnerable population, thus there is a need for transparency about interventions for IVF, including uncertainties and risks, to support patient decision-making regarding the use of certain adjunctive therapies. Such information can be provided by clear guidelines and effective regulation.

## TEXT

Despite the low delivery per fresh embryo transfer rate, over the past 40 years, the use of *in vitro* fertilization (IVF) has resulted in the birth of more than 9 million children around the world (Lancaster and de Mouzon 2019). IVF has been shown to be an extremely lucrative “business” that has revolutionized human reproduction by offering the hope of parenthood to couples who had been presumably defined as infertile.

The low success rate impacts the psychological well-being of patients, who routinely pay large sums of money for treatment and may be willing to try anything to improve their success rates (Harper et al. 2017). This has encouraged assisted reproduction centres to offer clinical adjuncts, more often known as add-ons, to infertile couples undergoing IVF with the intention of improving their chance of a live birth. These therapies may include laboratory techniques, clinical procedures or even the use of some medications, and their use appears to be widespread.

The problem is that there is no clear regulation for the use of such therapies. Requirements for introducing treatments into practice are relatively limited (van de Wiel et al. 2020) and do not include evidence of effectiveness and safety in randomized controlled trials. In the UK, before an adjunct treatment is offered, the legislation requires clinics to provide open and honest information about the existence of robust evidence to support the intervention, along with information about costs. The obligation for informed consent by the patient for adjunct treatment in IVF may be insufficient to eliminate the overselling or misselling of adjunct therapies with poor or even no scientific evidence of efficacy (Harper et al. 2017). Indeed, even though the lack of scientific evidence may be thoroughly explained to the patients, different explanations for reproductive failure circulate online and in the popular press, making patients prone to believing in “magic formulas”. This, associated with the emotional load inherent to infertility treatments and the high pressure put on both patients and their doctors, encourages patients to choose the method that they feel is most suitable for their needs.

A major concern emerges not only when social media or the popular press promote the use of IVF adjunct therapies but also when it is promoted by clinical websites. The promotion and provision of add-on treatments with a limited evidence base is common. In fact, many clinics around the world are offering these therapies to infertile couples undergoing IVF; however, the information available to patients from IVF clinic websites is often inaccurate. In a previously published report, different infertility clinic websites were examined to determine which of these advertised add-ons. It was concluded that add-ons were commonly offered in the context of self-funded treatment, which is a reality in a large part of most countries. Additionally, it was observed that very few websites stated that the effectiveness of the add-on was in doubt or unclear, and none raised the possibility that an add-on might have negative effects (van de Wiel et al. 2020). In another recently published article, 254 websites were evaluated, and 78.8% offered an accurate description of the provided add-ons; however, only a minority (12%) reported their undetermined effectiveness. The cost was not often presented (6.9%), and scientific evidence was only rarely provided. Additionally, none of the websites reported the clinic’s pregnancy rate following the add-on procedures (Galiano et al. 2021).

In a study performed in Australia and New Zealand, add-ons were advertised on 78% of the evaluated website clinic websites. In 77% of the cases, descriptions of the IVF add-ons were accompanied by claims of benefit; however, 90% of the claims were not quantified, and very few referenced scientific publications to support the claims. None of the add-ons were supported by high-quality evidence of a benefit for pregnancy or live birth rates (Lensen et al. 2021).

One may believe that the primary drive behind the use of unproven add-on treatments is not to improve outcomes for patients but to increase the profitability of the clinics offering them (Harper et al. 2017, Macklon et al. 2019). Howard (2018) described add-ons such as endometrial scratching, assisted hatching, or embryo glue, which are not regulated by the Human Fertilisation and Embryology Authority (HFEA), lack strong evidence of efficacy, and cost up to £3500. Nevertheless, this point of view proves to be a delicate issue for infertility clinicians.

Clearly, the use of add-ons involves ethical issues, which have been discussed at length (Armstrong et al. 2019, Kamath et al. 2019, Lensen et al. 2019, van de Wiel et al. 2020, Galiano et al. 2021, Lensen et al. 2021, Stein and Harper 2021, Liperis et al. 2022). While IVF clinic websites provide valuable information for patients seeking fertility treatment, this information is often not accurate nor complete.

More often than not, after an add-on has been introduced into routine practice, it will be found ineffective in randomized trials and, in some cases, even found to be harmful (Wilkinson et al. 2018). Gamete intrafallopian transfer, zygote intrafallopian tube transfer, and preimplantation genetic screening on a subset of chromosomes using fluorescence *in situ* hybridization have all proven inefficient (Armstrong et al. 2019). There is a need for clear information on interventions, including uncertainties and risks, to be made available by IVF clinics to support well-informed treatment.

This situation is especially critical in countries where IVF treatments are not subsidized by national health services. In countries such as the Netherlands, where IVF is almost entirely supported by health insurance providers, add-ons are not generally available, and the debate on their appropriate use is moved by commercial matters (Macklon et al. 2019).

The emotional and financial loads of undergoing fertility treatments can be high. The flood of information provided by websites, social media, popular press, etc., perpetuate false myths among this vulnerable population. According to a recently published study, in which an online survey was performed, 82% of women having IVF in the last 3 years had used one or more IVF add-on during treatment, usually at an additional cost (Lensen et al. 2021).

**Figure 1:**
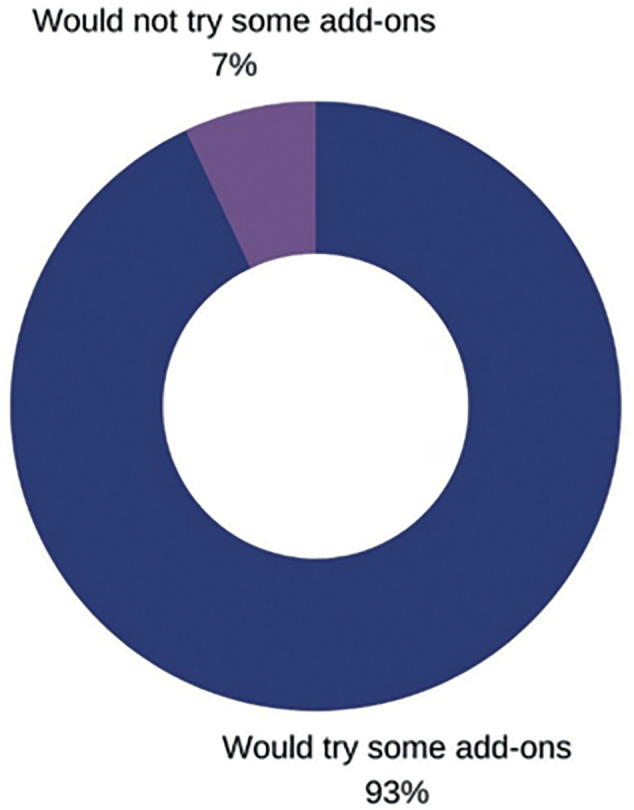
Patient's intention to try IVF add-ons: nonpublished data.

A nonpublished study by our group analysed the reported intentions to use add-ons among infertile patients (n=620) through participation in an online-platform survey. Survey results demonstrated that most patients (Figure 1) would try add-ons to increase their chances of success, even with no scientific evidence. Among those, most of them would try it at the beginning of the treatment (76.5%), while 23.5% of patients would try it only if they had a negative result first. When the answers of patients who were yet to start their treatments were compared with those who had already started treatment, we observed that those who were already involved in the treatment process were more willing to try add-ons when compared with those who had not yet started their treatments (97.0% *vs.* 84.1%, p<0.001). These results show that add-ons are well accepted by most infertile patients, especially those who have already started their IVF treatments.

Nevertheless, patients are not in a position to make clear decisions about which procedures to choose; therefore, a more effective regulation of add-ons is crucial. It is imperative that IVF clinic websites communicate the associated risks and uncertainties of add-ons to prospective patients.

In the UK, the HFEA has been prompted to release official guidelines on the effectiveness and safety of commonly offered add-ons. The document sought to guide both professionals and patients on the principles to be followed when deciding whether to utilize an add-on therapy with IVF treatment (HFEA 2020). In this document, fertility care providers are reminded to “offer fertility treatments ethically” and to adopt a “culture change” that, it is implied, protects patients from potential exploitation. The guidelines describe nine add-on treatments that were graded using a “traffic light system”, but no intervention was given the “green light”.

IVF add-ons continue to be offered and implemented in clinical practice before their safety is thoroughly ascertained. However, patients continue to request and pay large sums for such additional IVF tools. Questions remain as to whether it is ethical to provide IVF add-ons when there is no evidence of a benefit if the patient requests it. As described quite elegantly by Zemyarska (Zemyarska 2019), the issue involves key values of medical ethics-autonomy, beneficence and nonmaleficence. It was determined that providing IVF add-ons might be morally acceptable in specific circumstances: if true informed consent can be given, if there is a potential of cost-effective physiological or psychological benefit, and if the risk of harm is minimal, particularly concerning the unborn child.

Scientific efforts should continue to assess the effectiveness, safety and clinical relevance of both novel and preexisting adjunct IVF therapies and interventions. In the meantime, guidelines and clear legislation to regulate adjunct IVF tools should be created.
